# Kriterien für die Weiterbildungsbefugnis zur Facharztkompetenz Innere Medizin und Rheumatologie – ein Positionspapier der Deutschen Gesellschaft für Rheumatologie und Klinische Immunologie

**DOI:** 10.1007/s00393-024-01598-5

**Published:** 2024-12-12

**Authors:** Alexander Pfeil, Martin Fleck, Martin Aringer, Xenofon Baraliakos, Diana Ernst, Isabell Haase, Christiana Hillebrecht, Bimba Franziska Hoyer, Gernot Keyßer, Ina Kötter, Andreas Krause, Martin Krusche, Hanns-Martin Lorenz, Fabian Proft, Florian Schuch, Diana Vossen, Anna Voormann, Ulf Wagner, Jürgen Wollenhaupt, Christof Specker, Vorstand der Deutschen Gesellschaft für Rheumatologie und Klinische Immunologie, Kommission Fort- und Weiterbildung der Deutschen Gesellschaft für Rheumatologie und Klinische Immunologie

**Affiliations:** 1 Klinik für Innere Medizin III, Rheumazentrum (G-BA-Kriterien) sowie Sektion Rheumatologie und Osteologie, Universitätsklinikum Jena, Friedrich-Schiller-Universität Jena, Am Klinikum 1, 07747 Jena, Deutschland; 2 Klinik und Poliklinik für Innere Medizin I, Universitätsklinikum Regensburg, Regenburg, Deutschland; 3 Klinik und Poliklinik für Rheumatologie/Klinische Immunologie, Asklepios Klinikum Bad Abbach, Bad Abbach, Deutschland; 4 Bereich Rheumatologie, Medizinische Klinik und Poliklinik III, Universitätsklinikum „Carl Gustav Carus“ der Technischen Universität Dresden, Dresden, Deutschland; 5 Rheumazentrum Ruhrgebiet, Ruhr-Universität Bochum, Herne, Deutschland; 6 Klinik für Rheumatologie und Immunologie, Medizinische Hochschule Hannover, Hannover, Deutschland; 7 Sektion für Rheumatologie und Entzündliche Systemerkrankungen, Universitätsklinikum Hamburg-Eppendorf (UKE), Hamburg, Deutschland; 8 Klinik für Internistische Rheumatologie, Rotes Kreuz Krankenhaus, Bremen, Deutschland; 9 Sektion Rheumatologie, Klinik für Innere Medizin I, Universitätsklinikum Schleswig-Holstein, Kiel, Deutschland; 10 Department für Innere Medizin, Klinik für Innere Medizin II, Universitätsklinikum Halle, Halle (Saale), Deutschland; 11 Klinik für Innere Medizin, Abteilung Rheumatologie, klinische Immunologie und Osteologie, Immanuel Krankenhaus Berlin, Berlin, Deutschland; 12 Sektion Rheumatologie, Medizinische Klinik V, Universitätsklinikum Heidelberg, Heidelberg, Deutschland; 13 Abteilung für Rheumatologie, Medizinische Klinik für Gastroenterologie, Infektiologie und Rheumatologie, Campus Benjamin Franklin, Charité Universitätsmedizin, Berlin, Deutschland; 14 Internistische Praxisgemeinschaft Rheumatologie – Nephrologie, Erlangen, Deutschland; 15 Deutsche Gesellschaft für Rheumatologie und Klinische Immunologie, Berlin, Deutschland; 16 Bereich Rheumatologie, Klinik und Poliklinik für Endokrinologie, Nephrologie, Rheumatologie, Universitätsklinikum Leipzig, Leipzig, Deutschland; 17 Immunologikum Hamburg, Hamburg, Deutschland; 18 Klinik für Rheumatologie und Klinische Immunologie, Evangelisches Krankenhaus Kliniken Essen-Mitte, Essen, Deutschland

**Keywords:** Musterweiterbildungsordnung, Weiterbildungsinhalte, Weiterbildungsplan, Facharztweiterbildung, Facharztbezeichnung, Model training regulations, Training content, Training plan, Medical specialist training, Medical specialist qualification

## Abstract

Die Musterweiterbildungsordnung definiert die Weiterbildungsinhalte zur Erlangung der Facharztbezeichnung Innere Medizin und Rheumatologie. Kriterien zur Erteilung der Weiterbildungsbefugnis liegen nicht vor. Das Positionspapier beschreibt die von der Deutschen Gesellschaft für Rheumatologie und Klinische Immunologie (DGRh) vorgeschlagenen Kriterien, die bei der Erteilung einer Weiterbildungsbefugnis im Gebiet Innere Medizin und Rheumatologie und für die Bemessung ihres zeitlichen Umfangs zugrunde gelegt werden sollten. Dabei fungieren die Musterweiterbildungsordnung 2018 und der fachlich empfohlene Weiterbildungsplan als Basis. Anhand der Kriterien kann die Weiterbildungsbefugnis zur Facharztweiterbildung Innere Medizin und Rheumatologie standardisiert, abgestuft und transparent im gesamten Bundesgebiet vergeben werden. So wird eine qualitätsoptimierte Weiterbildung in der Rheumatologie ermöglicht, die an zukünftige Entwicklungen des Faches angepasst werden kann.

## Abstract (en)

The model advanced training regulations define the content of advanced training to achieve the qualification of medical specialist in internal medicine and rheumatology. There are currently no criteria for issuing the authorization in advanced training. This position paper describes the criteria proposed by the German Society for Rheumatology and Clinical Immunology (DGRh), which should be the foundation for the issuance of authorization for advanced training in the field of internal medicine and rheumatology and for the assessment of the duration. The model advanced training regulations 2018 and the advanced training plan recommended by experts function as the basis for this. Based on the criteria, the authorization for advanced training to advanced specialist training in internal medicine and rheumatology can be allocated in a standardized, graded and transparent manner throughout Germany. This enables an optimal quality of advanced training in rheumatology, which can be adapted to the future developments in the discipline.

## Einleitung

Das Fachgebiet der Rheumatologie beschäftigt sich mit Systemerkrankungen, welche durch eine immunvermittelte akute bzw. chronisch rezidivierende Entzündung im Bereich des Bewegungsapparates und verschiedener Organsysteme charakterisiert sind [2]. Darüber hinaus behandeln Rheumatologinnen und Rheumatologen auch nicht entzündliche rheumatische und muskuloskeletale Erkrankungen [13], wobei in Deutschland aufgrund der vorhandenen Kapazitäten der internistischen Rheumatologie der Fokus auf der Behandlung von entzündlich rheumatischen Erkrankungen liegt und das Positionspapier von der Situation in Deutschland ausgeht.

Unter Berücksichtigung der demografischen Entwicklung in Deutschland ist mit einem Anstieg der Inzidenz und Prävalenz von entzündlich rheumatischen Erkrankungen in den nächsten Jahren zu rechnen [1]. Aufgrund des bereits vorhandenen Mangels an Fachärzten im ambulanten und stationären Sektor [2] sowie des bevorstehenden Generationswechsel in der Rheumatologie [6] ist die erfolgreiche Weiterbildung zu Fachärztinnen bzw. Fachärzten für Innere Medizin und Rheumatologie für eine optimale Patientenversorgung unerlässlich.

Die Weiterbildungsinhalte zum Erwerb der Facharztbezeichnung Fachärztin/Facharzt für Innere Medizin und Rheumatologie basieren auf der Musterweiterbildungsordnung (MWBO) und der Konkretisierung der Weiterbildungsinhalte durch den fachlich empfohlenen Weiterbildungsplan (FEWP) [4, 5]. Entsprechend der Musterweiterbildungsordnung 2018 müssen mindestens 72 Monate im Fachgebiet der Inneren Medizin abgeleistet werden, wobei 36 Monate Weiterbildungszeit für das Fachgebiet Innere Medizin und Rheumatologie vorgesehen sind. Hierbei sind nach der MWBO mindestens 24 Monate in der stationären Patientenversorgung abzuleisten [4]. Von dieser Regelung wurde allerdings bei der Umsetzung in einzelnen Landesärztekammern abgewichen. Dies wurde durch den Mangel an stationären Weiterbildungsstellen begründet, was zu einem Engpass für die Weiterbildungsmöglichkeiten zum Rheumatologen führen könnte [2].

**Abb. 1:**
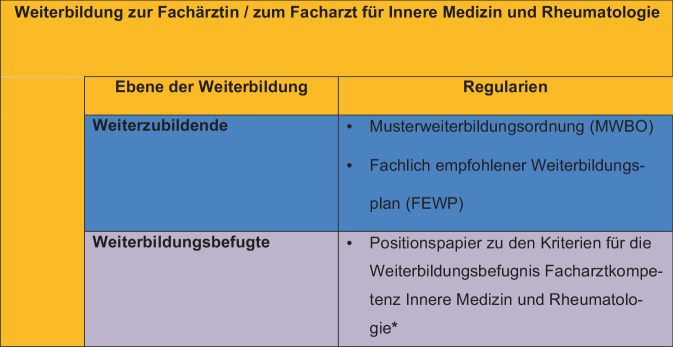
Ebenen der Weiterbildung zur Fachärztin bzw. zum Facharzt für Innere Medizin und Rheumatologie mit Darstellung der Regularien (*Sternchen:* zukünftige Einordnung des Positionspapiers der Kriterien für die Weiterbildungsbefugnis Facharztkompetenz Innere Medizin und Rheumatologie)

Die MWBO und der FEWP definieren detailliert die Kompetenzen und Weiterbildungsinhalte zur Erlangung der Facharztbezeichnung Innere Medizin und Rheumatologie auf der Ebene der Weiterzubildenden. Im Gegensatz hierzu wird die Qualifikation der Weiterbildungsbefugten nicht konkretisiert, sodass bisher keine Empfehlungen definiert sind, anhand welcher die Erteilung und der Umfang (12 Monate, 24 Monate bzw. 36 Monate) der Weiterbildungsbefugnis für das Fachgebiet Innere Medizin und Rheumatologie vorgenommen werden kann (s. Abb. 1).

Im Rahmen dieses Positionspapiers werden deshalb Kriterien zur Erteilung der Weiterbildungsbefugnis für das Fachgebiet Innere Medizin und Rheumatologie vorgeschlagen, die den Prozess der Befugniserteilung transparent bzw. standardisiert gestalten und die Qualität der Weiterbildung sichern sollen.

## Kriterien für die Weiterbildungsbefugnis Facharztkompetenz Innere Medizin und Rheumatologie

Bezogen auf die Erteilung einer Weiterbildungsbefugnis wurden die nachstehenden Befugniskriterien erarbeitet. Als Grundlage der Kriterien dienen die in der MWBO genannten Weiterbildungsblöcke und der FEWP [4, 5]. Hierbei stellen die Kriterien zur Erteilung der Weiterbildungsbefugnis entsprechende Mindestanforderungen an die Weiterbildungsbefugten dar.

### I. Zeitlicher Rahmen

Die zeitliche Grundlage für die Kriterien zur Erteilung der Weiterbildungsbefugnis der Facharztkompetenz Innere Medizin und Rheumatologie bildet die MWBO [4]. Zur Erteilung der Facharztkompetenz Innere Medizin und Rheumatologie müssen 72 Monate im Gebiet Innere Medizin absolviert werden, wovon 36 Monate auf das Fachgebiet Innere Medizin und Rheumatologie entfallen [4]. Im Weiteren beziehen sich die Kriterien für die Weiterbildungsbefugnis der Facharztkompetenz Innere Medizin und Rheumatologie auf den 36-monatigen Abschnitt im Fachgebiet Innere Medizin und Rheumatologie.

### II. Konkretisierung der Weiterbildungsinhalte (s. Abb. 2)

Die Konkretisierung der Weiterbildungsinhalte wird anhand der MWBO 2018 und des FEWP vorgenommen. Hierbei wird jedem konkreten Weiterbildungsinhalt eine Kompetenznummer zugeordnet, welche durch die Weiterbildungsbefugten vermittelt werden muss. Von den so entwickelten 33 Kompetenzen werden die Weiterbildungsinhalte mit den Kompetenznummern 3, 8 und 30 als obligat für eine volle rheumatologische Weiterbildungsbefugnis über 36 Monate eingestuft. Die Differenzierung in eine 12-monatige, 24-monatige oder volle Weiterbildungsbefugnis erfolgt unter Berücksichtigung der vorhandenen Kompetenzen an der Weiterbildungsstätte.

**Abb. 2:**
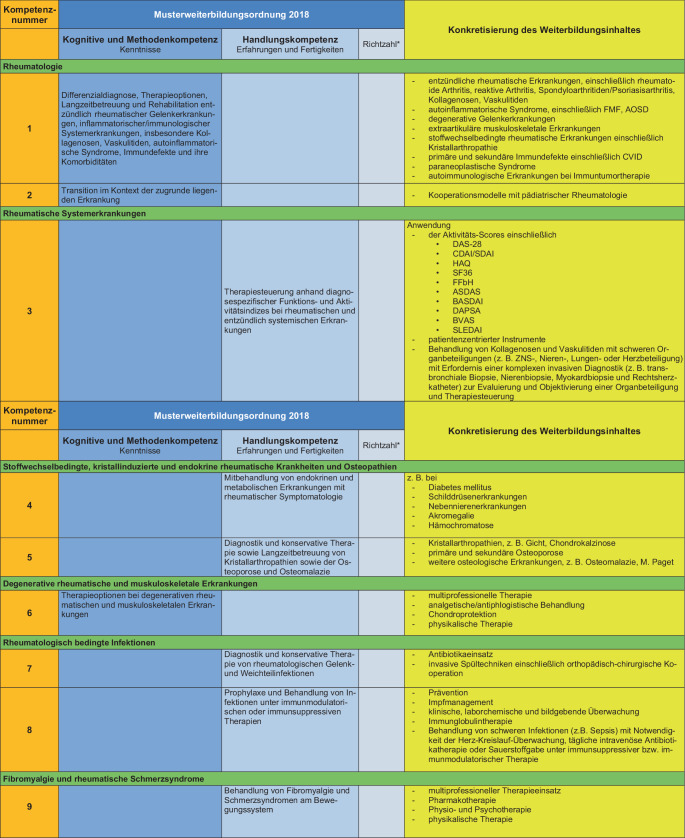
Konkretisierung der Weiterbildungsinhalte unter Bezug auf die spezifischen Inhalte der Facharztweiterbildung Innere Medizin und Rheumatologie. *Sternchen:* Auf die Nennung von Richtzahlen wird in diesem Positionspapier bewusst verzichtet, diese können ggf. herangezogen werden, um die Anzahl der Weiterbildungsstellen an einer Weiterbildungsstätte festzulegen

**Abb. 2:**
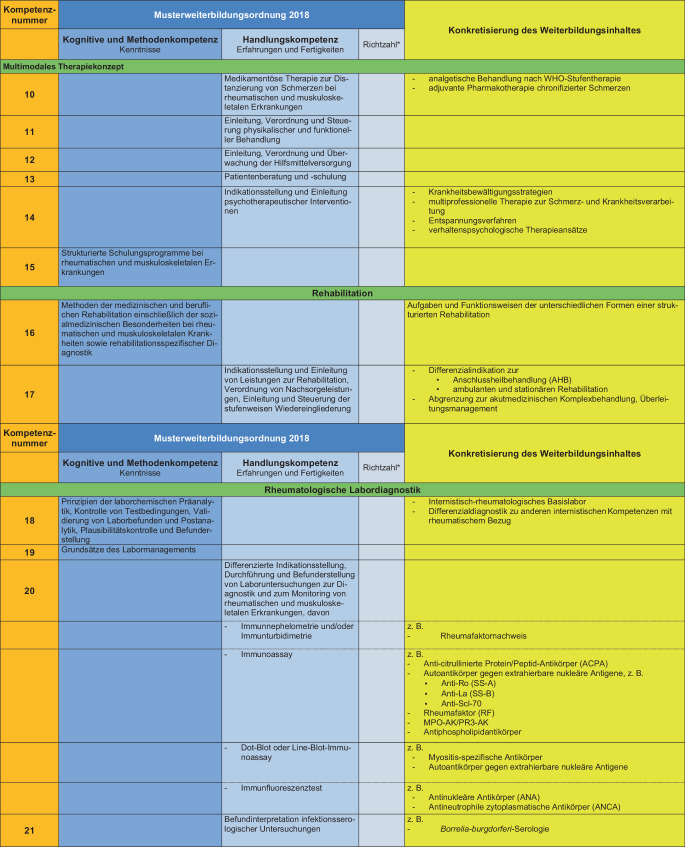
(Fortsetzung)

**Abb. 2:**
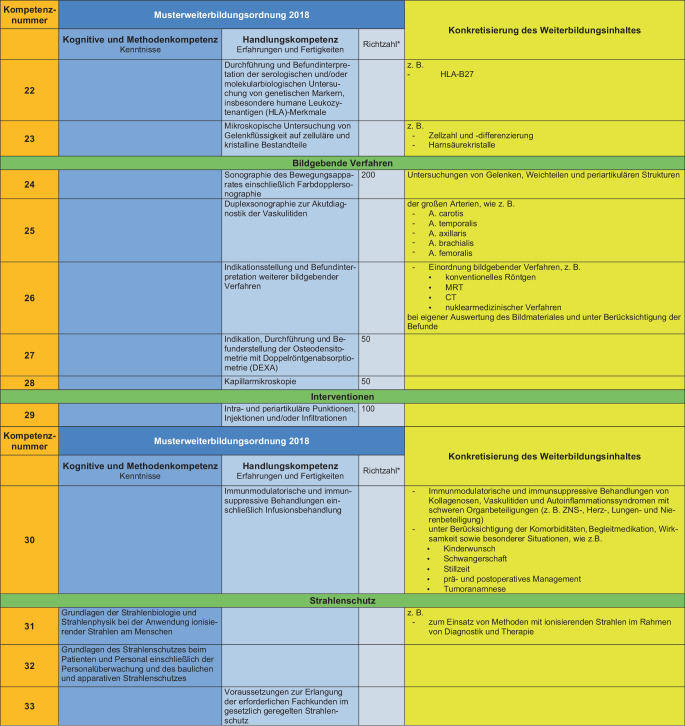
(Fortsetzung)

### III. Mindestkriterien für die spezifischen Inhalte der Facharztweiterbildung Innere Medizin und Rheumatologie (s. Tab. 1)

Können alle 33 Kompetenzen (Details s. Abb. 2) erfüllt werden, sollte eine Befugnis über 36 Monate erteilt werden. Werden 25 von 33 Kompetenzen erfüllt, wobei die Kompetenz mit der Nummer 30 als obligat anzusehen ist, kann eine Weiterbildungsbefugnis für 24 Monate erteilt werden. Eine Weiterbildungsbefugnis über 12 Monate wäre bei mindestens 20 von 33 Kompetenzen angemessen.

**Tab. 1:** Mindestkriterien für die spezifischen Inhalte der Facharztweiterbildung Innere Medizin und Rheumatologie

Umfang	Inhalt
36 Monate	**Alle 33 Kompetenzen erfüllt** **Kompetenzen 3, 8 und 30 obligat:** *Kompetenz 3:* Behandlung von Kollagenosen und Vaskulitiden mit schweren Organbeteiligungen (z. B. ZNS-, Nieren‑, Lungen- oder Herzbeteiligung) mit Erfordernis einer komplexen invasiven Diagnostik (z. B. transbronchiale Biopsie, Nierenbiopsie, Myokardbiopsie und Rechtsherzkatheter) zur Evaluierung und Objektivierung einer Organbeteiligung und Therapiesteuerung *Kompetenz 8:* Behandlung von schweren Infektionen (z. B. Sepsis) mit Notwendigkeit der Herz-Kreislauf-Überwachung, tägliche intravenöse Antibiotikatherapie oder Sauerstoffgabe unter immunsuppressiver bzw. immunmodulatorischer Therapie *Kompetenz 30:* Immunmodulatorische und immunsuppressive Behandlungen von Kollagenosen, Vaskulitiden und Autoinflammationssyndromen mit schweren Organbeteiligungen (z. B. ZNS-, Herz‑, Lungen- und Nierenbeteiligung)
24 Monate	25 von 33 Kompetenzen erfüllt Kompetenz 30 obligat
12 Monate	20 von 33 Kompetenzen erfüllt
*Die Graduierung des Weiterbildungsumfanges erfolgte im Konsens durch die Kommission Fort- und Weiterbildung der Deutschen Gesellschaft für Rheumatologie und Klinische Immunologie*	

### Ermittlung der jährlichen Leistungszahlen

Für die Erteilung der Weiterbildungsbefugnisse sind Leistungszahlen von besonderer Bedeutung und werden von den Landesärztekammern bewertet. Die Leistungszahlen bzw. Behandlungsfälle einer/eines Weiterbildungsbefugten werden üblicherweise nach folgender Formel berechnet:

$$\begin{aligned} &\frac{ \begin{array}{c} \text{J{\"a}hrliche Fallzahl/{}Leistungszahl}\\ \text{der/des Befugten} \end{array}}{\text{Anzahl der Weiterzubildenden}}\\[2mm] ={}&\text{j{\"a}hrlich erbrachte Leistungszahl je}\\ &\text{Weiterzubildenden} \end{aligned}$$

Auf detaillierte Leistungszahlen wird in diesem Positionspapier bewusst verzichtet, diese können ggf. herangezogen werden, um die Anzahl der Weiterbildungsstellen an einer Weiterbildungsstätte festzulegen.

### Zuordnung der Weiterbildungskompetenz (s. Abb. 3)

Für den Umfang der zu erteilenden Weiterbildungsbefugnis ist maßgebend, inwieweit die an Inhalt, Ablauf und Zielsetzung der Weiterbildung gestellten Anforderungen durch die Weiterbildungsbefugten unter Berücksichtigung des Versorgungsauftrages, der Leistungsstatistik sowie der personellen und materiellen Ausstattung der Weiterbildungsstätte erfüllt werden können. Hieraus ergeben sich die an der Weiterbildungsstätte zu vermittelnden Kompetenzen, welche nach Umfang und inhaltlicher Abstufung einer Erteilung der Weiterbildungsbefugnis dienen.

## Diskussion

Anhand der Musterweiterbildungsordnung 2018 wurden für die Weiterzubildenden im Fachgebiet Innere Medizin und Rheumatologie von den Landesärztekammern kammerspezifische Weiterbildungsordnungen zur Erlangung der Fachärztin/des Facharztes für Innere Medizin und Rheumatologie entwickelt [4]. Die Inhalte der MWBO zur Weiterbildung werden im FEWP konkretisiert, der den Weiterzubildenden sowie den Weiterbildungsbefugten eine Orientierung für den Kompetenzerwerb gibt [4, 5]. Mit dem 2021 veröffentlichten Mustercurriculum der Deutschen Gesellschaft für Rheumatologie und Klinische Immunologie (DGRh) für die Weiterbildung im Fachgebiet Innere Medizin und Rheumatologie wurde ein Leitfaden für die standardisierte Vermittlung von Kernkompetenzen im Rahmen der Facharztweiterbildung für Innere Medizin und Rheumatologie geschaffen [10]. Im Gegensatz dazu liegen für die Weiterbildungsbefugten bisher keine standardisierten Kriterien hinsichtlich der Erteilung der Weiterbildungsbefugnis vor. Mit der Veröffentlichung dieses Positionspapieres zur Weiterbildungsbefugnis werden erstmalig Kriterien zur Erteilung der Weiterbildungsbefugnis für das Fachgebiet Innere Medizin und Rheumatologie seitens der Fachgesellschaft definiert. Der vorgestellte Kriterienkatalog eröffnet die Möglichkeit einer transparenten und anhand von vermittelten Kompetenzen abgestuften Erteilung der Weiterbildungsbefugnis für das Fachgebiet Innere Medizin und Rheumatologie und hilft, die Qualität der Weiterbildung zu sichern.

Als Basis zur Etablierung von Kriterien zur Erteilung der Weiterbildungsbefugnis wurden 33 Kompetenzen definiert mit Konkretisierung des Weiterbildungsinhaltes auf Basis der MWBO und des FEWP. Es wurden obligate Kompetenzen festgelegt, und auf der Basis der an der Weiterbildungsstätte vorhandenen Kompetenzvermittlung wurde eine Graduierung der Weiterbildungsbefugnisse über 12 bis 36 Monate entwickelt.

Bislang findet die Weiterbildung zur Rheumatologin bzw. zum Rheumatologen in Deutschland überwiegend im stationären Sektor statt [9], wobei die stationäre Weiterbildung in verschiedenen Klinikformen (Universitätskliniken, nichtuniversitäre Kliniken und Rehabilitationskliniken) und demzufolge an unterschiedlichen Patientenkollektiven mit differenter Ausprägung rheumatischer Krankheitsbilder erfolgt. Der Möglichkeit einer Weiterbildung auch im niedergelassenen Bereich wird durch den vorgestellten kompetenzbasierten Kriterienkatalog auch in einer heterogenen Versorgungs- und Weiterbildungsstruktur in der Rheumatologie Rechnung getragen. Dies ermöglicht, transparent und unabhängig von der Versorgungsstruktur die Möglichkeiten der Kompetenzvermittlung für jede Weiterbildungsstätte zu beurteilen. Auch lässt der vorgelegte Kriterienkatalog rasche Rückschlüsse bezüglich fehlender Kompetenzen der Weiterbildungsstätte zu, die durch entsprechende Kooperationen von den Weiterzubildenden an einer anderen Weiterbildungsstätte zu erwerben sind. Diesbezüglich erleichtert der vorliegende Kriterienkatalog auch die Einrichtung von Kooperationen verschiedener Weiterbildungsbefugten über die verschiedenen Sektoren der Patientenversorgung hinweg und ermöglicht die Etablierung strukturierter Verbundweiterbildungsprogramme. Dies ist auch im Hinblick, auf den bestehenden Facharztmangel sowie den anstehenden Generationswechsel dringend notwendig [2, 6, 8], um die vorhandenen ambulanten und die verschiedenen stationären Weiterbildungskapazitäten optimal zu nutzen [11]. Dieses Vorgehen trägt auch der auf Bundesebene angestrebten sektorübergreifenden Versorgung von Patientinnen und Patienten hinsichtlich der Weiterbildung Rechnung.

Zur Graduierung der Weiterbildungsbefugnis wurden entsprechende Kompetenzen sowie in der Ausprägung differente entzündlich rheumatische Erkrankungen unter Berücksichtigung von Organbeteiligungen und Komplikationen in den Kriterien hinterlegt, um die Ausbildungsqualität über die gesamte Bandbreite entzündlich rheumatischer Erkrankungen an den Weiterbildungsstätten sicherzustellen. Hierdurch wird auch das Interesse der Weiterzubildenden berücksichtigt, die eine Kompetenzvermittlung durch strukturierte Aus- und Weiterbildungsprogramme fordern [7, 12].

Die DGRh strebt eine kontinuierliche Weiterentwicklung des Kriterienkatalogs für die Weiterbildungsbefugnis der Facharztkompetenz Innere Medizin und Rheumatologie an. Die Kommission Fort- und Weiterbildung nimmt Kommentare bzw. Änderungsvorschläge hierzu jederzeit gerne entgegen. Außerdem müssen in Zukunft die Kriterien zur Erteilung der Weiterbildungsbefugnis aufgrund der diagnostischen und therapeutischen Weiterentwicklungen im Fachgebiet Innere Medizin und Rheumatologie kontinuierlich angepasst werden.

Unter Berücksichtigung des bevorstehenden Generationswechsels in der Rheumatologie als auch des Facharztmangels ist es von besonderer Wichtigkeit [2, 3, 6], über eine attraktive Weiterbildung motivierte junge Ärztinnen und Ärzte für das Fachgebiet zu gewinnen. Hierbei stellen das Mustercurriculum der DGRh für die Weiterbildung im Fachgebiet Innere Medizin und Rheumatologie als auch der vorliegende Kriterienkatalog zur Erteilung der Weiterbildungsbefugnis Leitfäden dar, welche auf 2 unterschiedlichen Ebenen eine hoch qualitative und attraktive Weiterbildung im Fachgebiet Innere Medizin und Rheumatologie ermöglichen.

**Abb. 3:**
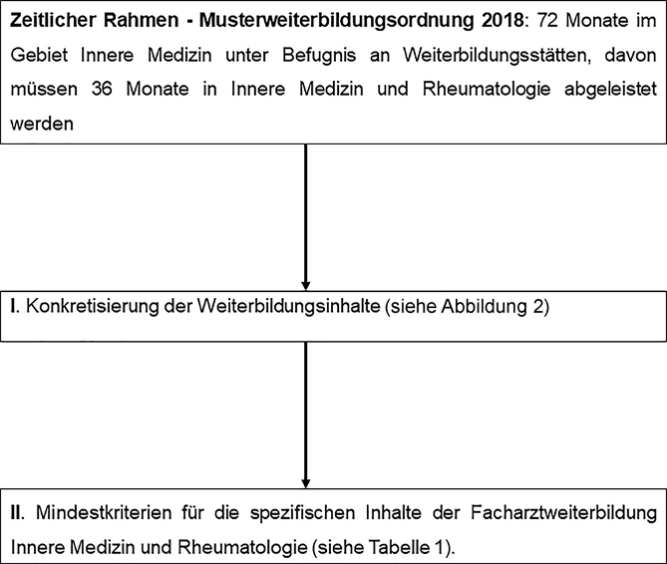
Flowchart für Kriterien zur Erteilung der Weiterbildungsbefugnis für die Facharztkompetenz Innere Medizin und Rheumatologie

## Abkürzungen

DGRh: Deutsche Gesellschaft für Rheumatologie und Klinische Immunologie
FEWP: Fachlich empfohlener Weiterbildungsplan
MWBO: Musterweiterbildungsordnung
